# Molecular Genetics and Functional Anomalies in a Series of 248 Brugada Cases with 11 Mutations in the TRPM4 Channel

**DOI:** 10.1371/journal.pone.0054131

**Published:** 2013-01-30

**Authors:** Hui Liu, Stéphanie Chatel, Christophe Simard, Ninda Syam, Laurent Salle, Vincent Probst, Julie Morel, Gilles Millat, Michel Lopez, Hugues Abriel, Jean-Jacques Schott, Romain Guinamard, Patrice Bouvagnet

**Affiliations:** 1 Laboratoire Cardiogénétique, Hospices Civils de Lyon, Groupe Hospitalier Est, Bron, France; 2 Laboratoire Cardiogénétique, Equipe d’Accueil 4173, Université Lyon 1, Lyon, France; 3 Service de Cardiologie Pédiatrique, Hospices Civils de Lyon, Groupe Hospitalier Est, Bron, France; 4 Unité Mixte de Recherche 915, Institut National de la Santé Et de la Recherche Médicale, l’institut du thorax, Nantes, France; 5 Equipe de Recherche Labellisée 3147, Centre National de la Recherche Scientifique, l’institut du thorax, Nantes, France; 6 Université de Nantes, l’institut du thorax, Nantes, France; 7 Service de Cardiologie, CHU Nantes, l’institut du thorax, Nantes, France; 8 Groupe Signalisation, Electrophysiologie et Imagerie des lésions d’ischémie- reperfusion myocardique, Equipe d’Accueil 4650, Université de Caen, Centre Hospitalier Universitaire de Caen, Caen, France; 9 Department of Clinical Research, University of Bern, Bern, Switzerland; 10 Service de Cardiologie, Hôpital Saint Luc, Saint Joseph, Lyon, France; 11 Laboratoire Neurocardiologie, EA 4612, Université Lyon 1, Lyon, France; University of Tampere, Finland

## Abstract

Brugada syndrome (BrS) is a condition defined by ST-segment alteration in right precordial leads and a risk of sudden death. Because BrS is often associated with right bundle branch block and the *TRPM4* gene is involved in conduction blocks, we screened *TRPM4* for anomalies in BrS cases. The DNA of 248 BrS cases with no *SCN5A* mutations were screened for *TRPM4* mutations. Among this cohort, 20 patients had 11 *TRPM4* mutations. Two mutations were previously associated with cardiac conduction blocks and 9 were new mutations (5 absent from ∼14′000 control alleles and 4 statistically more prevalent in this BrS cohort than in control alleles). In addition to Brugada, three patients had a bifascicular block and 2 had a complete right bundle branch block. Functional and biochemical studies of 4 selected mutants revealed that these mutations resulted in either a decreased expression (p.Pro779Arg and p.Lys914X) or an increased expression (p.Thr873Ile and p.Leu1075Pro) of TRPM4 channel. *TRPM4* mutations account for about 6% of BrS. Consequences of these mutations are diverse on channel electrophysiological and cellular expression. Because of its effect on the resting membrane potential, reduction or increase of TRPM4 channel function may both reduce the availability of sodium channel and thus lead to BrS.

## Introduction

Brugada syndrome (BrS) is characterized by ST-segment elevation in the right precordial leads (V1– V3) of the electrocardiogram (ECG) with an associate risk of cardiac arrhythmia [1]. The mean age of BrS clinical appearance is around 40 years with a strong male preponderance [2], [3]. The ECG signature of BrS is transient and can be unmasked by administration of sodium channel blockers such as ajmaline or flecainide [2], [4]. There are internationally accepted criteria to establish a diagnosis of BrS [5].

The prevalence is estimated to be approximately 1/2500. Although numerous environmental factors influence BrS clinical and ECG expressivity, it is commonly accepted that it is a genetic disease with usually an autosomal dominant pattern of inheritance [6], [7]. Since 1998, it has been established that about 15–25% of BrS cases can be linked to mutations in *SCN5A* that encodes the alpha subunit of cardiac sodium channel Nav1.5 [8]. Several other genes have been implied in BrS such as *GPD1L, CACNA1C*, *CACNB2*, *SCN1B*, *KCNE3, SCN3B*, *KCNJ8* [9], *CACNA2D1* [10], *KCND3* [11] and *MOG1* [12] (for a review see [13]).

The transient receptor potential melastatin protein number 4 (TRPM4) is a calcium-activated nonselective cation channel, member of a large family of transient receptor potential genes [14]. *TRPM4* has been recently implied in families with progressive cardiac conduction blocks [15], [16], [17]. In this study, we addressed the question whether BrS cases could be attributed to *TRPM4* mutations since BrS is frequently associated with cardiac conduction anomalies. In a large cohort of 248 BrS cases with no *SCN5A* mutation, 11 *TRPM4* mutations were found in 20 unrelated individuals. The electrophysiological and cellular expression consequences of 4 mutations were further studied. These findings suggest that *TRMP4* mutations accounts for about 6% of BrS.

## Materials and Methods

### Ethics

A signed informed consent was obtained from all participants (or the parents of minors) prior to history recording and blood drawing. This study was specifically approved by the local ethics committees (comité de protection des personnes Ouest IV and Sud-Est II) and is in accordance with the last version of the Declaration of Helsinki (The World Medical Association, 2002).

### Clinical Evaluation

The diagnosis of Brugada is based on a type 1 ECG at rest prior or after a drug challenge (ajmaline or flecainide). Medical history was recorded and a clinical cardiologic examination was performed on all patients. Most of the participants had additional examinations including echocardiogram, stress test, ambulatory ECG recording and electrophysiological study.

### Genetic Analysis

DNA was extracted from blood samples according to standard protocols. Mutation screening of *TRPM4* (RefSeq NM_017636.3, OMIM# 606936) was carried out by High Resolution Melting (HRM) analysis (Rotor-Gene Q, Qiagen, Courtaboeuf, France) followed by bi-directional sequencing of abnormal profiles or directly by sequencing. The primers were already published [16]. The Exome Variant Server ( http://evs.gs.washington.edu/EVS/ ) was used to add controls to our series. Variants were confirmed on a second sample and a second PCR product.

### Preparation of TRPM4 Mutants

The complete human wild-type TRPM4 cDNA was cloned in pcDNA4/TO vector (Invitrogen, Cergy Pontoise, France) [16]. Mutants were obtained by *in vitro* mutagenesis using QuickChange II site-directed mutagenesis kit (Agilent Technologies, Massy, France). Mutant cDNA clones were systematically resequenced before use in further experiments.

### Stable TRPM4 Mutant Expression

pcDNA4/TO plasmid containing the diverse TRPM4 mutants were used to transfect T-REx™ 293 cell lines with Lipofectamine 2000 (Invitrogen, Cergy Pontoise, France) according to manufacturer specifications. The T-REx™ 293 cell line stably expresses the tetracycline repressor protein enabling the silencing of the gene of interest unless tetracycline is added to the culture medium. T-REx™ 293 is a stable transformed cell line of HEK 293 obtained with a plasmid that encodes the Tet repressor under the control of the human CMV promoter. Several stable clones (3–4) of each TRPM4 mutant were obtained according to Invitrogen protocol by selecting with blasticidin (Tet repressor) and zeocin (TRPM4). These stable clones were used for the electrophysiological study.

### Electrophysiology

Currents were recorded from whole-cell or inside-out patches of T-RexTM 293 transfected cells with a patch-clamp amplifier Axopatch 200B (Axon instruments, Forster city, CA, USA) using pClamp 9 software (Axon instruments). Experiments were conducted at room temperature.

For patch-clamp experiments in inside-out conditions, cells were bathed in a solution containing (in mM): 140 NaCl; 4.8 KCl; 1.2 MgCl_2_; 0.1 CaCl_2_; 10 glucose; and 10 HEPES, pH 7.4 (with NaOH). Solutions perfused at the inside of the membrane contained the previous solution (with 1 mM CaCl_2_) or, for determination of ionic selectivity, a low NaCl solution (in mM): 42 NaCl; 1.2 MgCl_2_; 1 CaCl_2_; 10 glucose; and 10 HEPES, supplemented with sucrose, pH 7.2.

In the whole-cell condition, TRPM4 currents were investigated using a ramp protocol. The holding potential was −60 mV. The 400 ms increasing ramp from −100 to +100 mV ends with a 20 ms step at +100 mV. The measured current was then reported to cell size estimated by capacitance measurement. A new ramp was performed every 5 s. As previously reported [18], in this mode, the TRPM4 current develops with time after membrane break to stabilize within 10 minutes. Biophysical properties were then estimated after current stabilization. To investigate channels activation time, a pulse protocol was used from a holding potential at 0 mV to +80 mV during 100 ms.

For whole-cell recordings, pipette solutions contained (in mM) 156 CsCl, 1 MgCl_2_ and 10 HEPES (pH adjusted to 7.2 with CsOH and [Ca^2+^] 10^−6 ^M). Bath and perfused solutions contained (in mM) 156 NaCl, 5 CaCl_2_, 10 glucose and 10 HEPES (pH adjusted to 7.4 with NaOH).

### Biotinylation Assay

For biotinylation assay, HEK-293 cells were transiently transfected with 240 ng of either HA-TRPM4 WT or mutants cDNAs or the empty vector in a P100 dish (BD Falcon, Durham, North Carolina, USA) mixed with 100 ul of OPTI-MEM I, 1 ul of Plus reagent and 3 ul of Lipofectamine LTX (Invitrogen, Carlsbad, California, USA). The cells were incubated for 48 hours at 37°C with 5% CO_2_. Following 48 hours of incubation, HEK-293 cells transiently transfected with either HA-TRPM4 WT or mutant cDNAs or the empty vector were treated with EZ-link™ Sulfo-NHS-SS-Biotin (Thermo Scientific, Rockford, Illinois, USA) 0.5 mg/ml in cold PBS for 15 minutes at 4°C. Subsequently, the cells were washed twice with 200 mM Glycine in cold PBS and twice with cold 1XPBS to inactivate and remove the excess biotin, respectively. The cells were then lysed with 1× lysis buffer (50 mM HEPES pH 7.4; 150 mM NaCl; 1.5 mM MgCl_2_; 1 mM EGTA pH 8; 10% Glycerol; 1% Triton X-100; 1× Complete Protease Inhibitor Cocktail (Roche, Mannheim, Germany) for 1 hour at 4°C. Cell lysates were centrifuged at 16,000 g 4°C for 15 minutes. Two milligrams of the supernatant was incubated with 50 ul Streptavidin Sepharose High Performance beads (GE Healthcare, Uppsala, Sweden) for 2 hours at 4°C, and the remaining supernatant was kept as the input. The beads were subsequently washed five times with 1× lysis buffer before elution with 50 ul of 2× NuPAGE sample buffer (Invitrogen, Carlsbad, California, USA) plus 100 mM DTT at 37°C for 10 minutes. These biotinylated fractions were analyzed as TRPM4 expressed at the cell surface. The input fractions, analyzed as total expression of TRPM4, were resuspended with 4× NuPAGE Sample Buffer plus 100 mM DTT to give a concentration of 1 mg/ml and incubated at 37°C for 10 minutes.

### Western Blotting

Both input and biotinylated fractions were analyzed on 8% polyacrylamide gel and detected with anti-TRPM4 antibody raised against the C terminal portion of TRPM4 from amino-acids 1138 to 1156 (Pineda, Berlin, Germany) and anti-α-actin A2066 (Sigma, St. Louis, Missouri, USA) antibodies. The blots obtained were quantified using IGOR Pro (Wavemetrics, Lake Oswego, Oregon, USA) software.

### Statistics

Variant prevalence in the BrS vs control cohorts was tested by the Fisher exact test and one sided p values are presented in table 1. Mutant electrophysiological values and quantified bands on Western blots were compared to wild type values using a Student t test with a probability value below 0.05 considered as significant.

**Table 1:** Presentation of TRPM4 variants.

mRNA	Protein	Grantham [0-215]	Splicing	Interspecies conservation	InterTRPM conservation	Protein domain	Controls	European American	African American	Total 1	Fisher exact test	Total 2	Fisher exact test
c. 430C>T	p.R144W	101	–	mammals	0/7	N-term. Intracyto	0/2040	0/3490	0/1854	0/5530	0.0429*	0/7384	0.0325*
c. 1294G>A	p.A432T	58	–	vertebrates	7/7	N-term. Intracyto	0/300	0/3501	0/1864	0/3801	0.0612	0/5665	0.0419*
c. 1663G>A	p.G555R	125	–	mammals except rodent	0/7	N-term. Intracyto	0/2000	0/5366	0/3501	0/7366	0.0323*	0/10867	0.0245*
c. 1744G>A	p.G582S	56	crypticdonorsite	mammals	4/7	N-term. Intracyto	0/2000	0/5356	0/3495	0/7356	0.0326*	0/10851	0.0223*
c.2317T>A	p.F773I	21	–	primates	0/7	1st extra-cellular loop	0/2000	0/5219	0/3405	0/7219	0.0332*	0/10624	0.0228*
c.2336C>G	p.P779R	103	–	invertebrates	6/7	Transmembrane S2	0/2000	0/5219	0/3405	0/7219	0.0332*	0/10624	0.0228*
c. 2618C>T	p.T873I	89	–	several mammals	0/7	Transmembrane S3	0/2000	0/6974	0/3626	0/8974	0.0269*	0/12600	0.0193*
c.2209G>A	p.G737R	125	–	several mammals	0/7	1st extra-cellular loop	1/2000	7/7007	6/3732	8/9007	0.0279*	14/12739	0.0365*
c.2531G>A	p.G844D	94	–	mammals	0/7	1st intra-cellular loop	4/2000	12/6780	0/3626	16/8780	0.0143*	16/12406	0.0056*
c.2561A>G	p.Q854R	43	–	primates + fish	3/7	1st intra-cellular loop	2/2000	8/6966	1/3713	10/8966	0.0045*	11/12679	0.0022*
c.2740A>T	p.K914X	–	–	mammals + fish	4/7	End of S4	2/1914	12/7008	3/3735	14/8922	0.0100*	17/12657	0.0063*
c.3224T>C	p.L1075P	98	–	invertebrates	4/7	TRP domain	0/2052	1/7019	0/3738	1/9071	0.0525	1/12809	0.0376*
c.1458_1493del36	p.K487_L498del	–	–	primates	0/7	N-term. Intracyto	4/576				0.8322		
c.3611C>T	p.P1204L	98		vertebrates	0/7	C-term. Intracyto	6/1100	25/6957	0/3708	31/8057	0.6473	31/11765	0.4875

(*: Fisher exact test positive) TRPM4 RefSeq NM_017636.3, OMIM 606936.

## Results

### Study Subjects and *TRPM4* Screening

**Figure 1:**
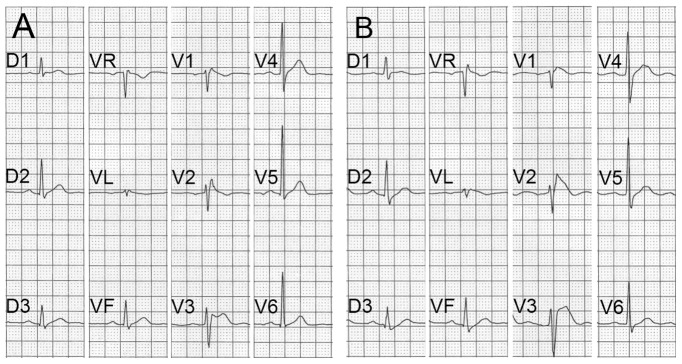
ECG of patient 9 with Brugada features. (**A**) Unchallenged and (**B**) ajmaline-challenged ECG of patient 9 showing a transition from Brugada type 2 to type 1. Note the characteristic ST segment elevation in V1, V2 and V3.

A cohort of 331 Brugada patients was studied. The diagnosis of BrS was based on a spontaneous or drug-challenged type 1 ECG pattern (figure 1A–B). All participants were screened for mutation in the gene encoding the alpha subunit of the sodium channel gene (*SCN5A*) and 83 patients (25%) had a *SCN5A* variant. These 83 patients were excluded for further study.

**Figure 2:**
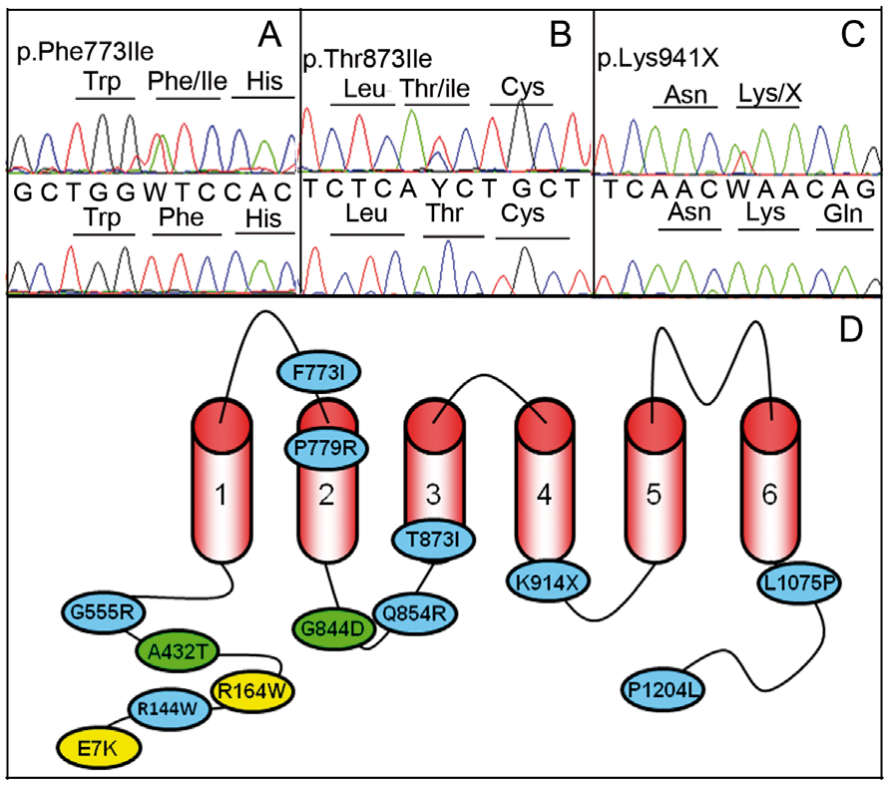
Electrophoregrams of 3 missense mutations and localization of TRPM4 mutations (A–C). Electrophoregrams of 3 mutations. W for A/T heterozygosity, and Y for C/T. (**D**): Localization of TRPM4 mutations resulting in conduction blocks (yellow), Brugada syndrome (blue) or both (green).

The screening of *TRPM4* in this cohort of 248 BrS cases with no *SCN5A* mutations evidenced 14 heterozygous variants in 25 unrelated individuals (figure 2 A–D). Five of these variants were considered as mutations on the ground that they changed conserved amino acids and were absent of our control series and among European Americans and African Americans controls of the Exome Variant Server (Table 1, Mutations). In addition, two variants (p.A432T and p.G844D) were previously reported in familial autosomal conduction block and their deleterious consequences demonstrated by familial segregation and experimental analysis [16]. Four variants were considered as putative genetic BrS predisposing factors on the ground that they changed conserved amino acids and they were found in the control population but the prevalence in the BrS cohort was statistically higher than in the control population (Table 1, Predisposing factors). Finally, 3 variants were considered as polymorphisms on the ground that they were found with a similar prevalence in the BrS cohort and the control populations. All the mutations and predisposing genetic factors were missense changes but we also observed a single non-sense variation in the predisposing factor sub-group (p.K914X). No patients had 2 mutations and/or predisposing genetic factors.

Table 2 summarizes the clinical data of the 20 *TRPM4* variant carriers. There were many more males than females (18/2). The average age at diagnosis was 49 years. The circumstances of discovery were in 9 cases a routine ECG, in 1 case a chest pain, in 9 cases an episode of fainting and in a single case a sudden death. Three had familial cases of sudden death and in one case the father’s patient experienced palpitations. Twelve patients had a spontaneous type 1 Brugada whereas 8 patients had a transition from type 2 to type 1 induced by drug challenge (ajmaline in 4 cases and flecainide in 4 cases). Only 2 patients (patients 10 and 23) had QRS duration shorter than 100 msec. Ten patients had an isolated incomplete RBBB whereas 3 had a isolated complete RBBB. Three patients had a bifascicular block. Finally, a single case (patient 13) had a prolonged QTc interval (458 ms).

**Table 2:** Presentation of clinical and electrocardiographic features of TRPM4 variant carriers.

Patientnumber	Mutations	Sex	Age at diagnostic	Circumstances of discovery	Family history	Spontaneous ECG	Pharmacology challenge	Heart rate	PR interval	QRS duration	Conduction blocks	QT	QTc
1	p.R144W	M	57	ECG	no	type 2	flecaïne +	86	138	110	iRBBB	340	407
2	**p.A432T**	M	36	ECG	no	type 2	flecaïne +	64	160	100	iRBBB	400	410
8	**p.G555R**	F	53	ECG	no	type 2	ajmaline +	84	166	104	–	340	402
9	**p.F773I**	M	52	chest pain	Father: palpitations	type 2	ajmaline +	80	160	100	iRBBB	320	388
11	**p.P779R**	M	57	ECG	no	type 1	–	72	170	100	iRBBB	364	399
10	**p.G844D**	M	45	ECG	SD	type 2	flecaïne +	69	166	79	rSr’	349	374
12	**p.G844D**	M	31	fainting	no	type 1	–	58	200	140	RBBB	420	415
13	**p.G844D**	M	56	fainting	SD	type 1	–	105	138	113	iRBBB	346	458
14	p.Q854R	M	45	fainting	no	type 2	flecaïne +	70	170	100	iRBBB	360	391
15	p.Q854R	M	55	fainting	no	type 1	–	73	180	120	RBBB	400	434
16	p.Q854R	F	50	fainting	no	type 1	–	53	281	106	AV block+iRBBB	455	423
17	**p.T873I**	M	34	ECG	no	type 1	–	61	220	120	AV block+RBBB	360	360
18	p.K914X	M	37	fainting	no	type 1	–	58	168	100	–	365	359
19	p.K914X	M	74	fainting	no	type 2	ajmaline +	50	150	120	RBBB+LAHB	480	440
20	p.K914X	M	35	ECG	SD	type 1	–	94	176	100	iRBBB	325	407
21	**p.L1075P**	M	44	ECG	no	type 1	–	58	168	105	iRBBB	372	366
22	p.I1204L	M	76	ECG	no	type 2	ajmaline +	70	180	130	RBBB	400	432
23	p.I1204L	M	43	fainting	no	type 1	–	54	193	87	rSr’	369	360
24	p.I1204L	M	70	SD	no	type 1	–	55	179	104	iRBBB	360	370
25	p.I1204L	M	35	fainting	no	type 1	–	73	172	107	iRBBB	352	388

AV block: AtrioVentricular block, iRBBB and RBBB: incomplete and complete Right Bundle Branch Block, LAHB: Left Anterior HemiBlock, QTc: corrected QT interval (Bazett formula). Mutations in bold, predisposing factors in regular letters. TRPM4 RefSeq NM_017636.3, OMIM 60.

Among the 5 new *TRPM4* mutations, 3 were selected for further electrophysiological and expression testing (P779R, T873I and L1075P). In addition, 1 variant among the predisposing factor sub-group, was also selected for further study: the mutation leading to a stop codon (K914X). These 3 missense mutations gave a Grantham score [19] of 89 or more. Grantham is a formula estimating difference between amino acid according to their physico-chemical properties. In addition, they were found in BrS patients with no other *TRPM4* variants (and no *SCN5A* variants), a situation resulting in a simpler correlation between phenotype and genotype.

### Expression of TRPM4 Variants

#### TRPM4 current detection in the whole-cell configuration

**Figure 3:**
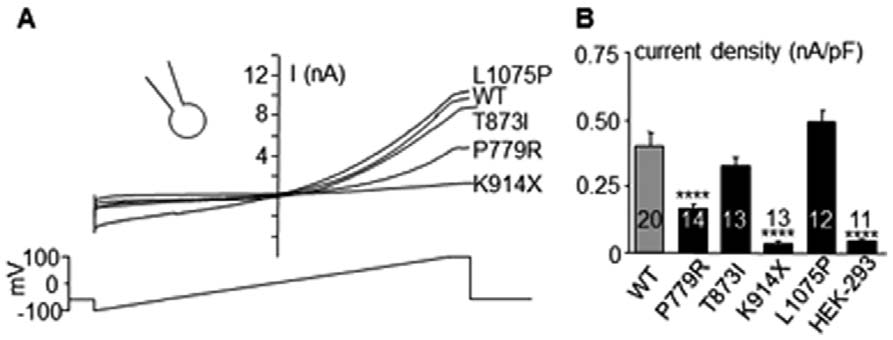
Biophysical properties of WT and mutants TRPM4 channel in whole-cell configuration. (**A**): Representative current tracings recorded in the whole cell conditions (ramp protocol under the traces) (**B**): Mean current density for WT and mutants estimated using the maximal current recorded during the ending step of 20 ms at Vm = +100 mV (see A) and reported to cell capacitance. *p<0.05, **p<0.01, ***p<0.001, ****p<0.0001, n.s.: not significant. Numbers in bars = number of experiments. Error bar: standard error of the mean.

Wild type (WT) TRPM4 and all mutants exhibited a characteristic outward rectifying current when recorded in the whole-cell configuration (figure 3 A).

A significant decrease in current density was detected for p.Pro779Arg and p.Lys914X transfected cells, in comparison to WT transfected cells (figure 3 B). The p.Lys914X transfected cells exhibited a current density similar to non-transfected HEK-293 cells, indicating that the mutant did not induce additional current.

#### Single channel conductance

**Figure 4:**
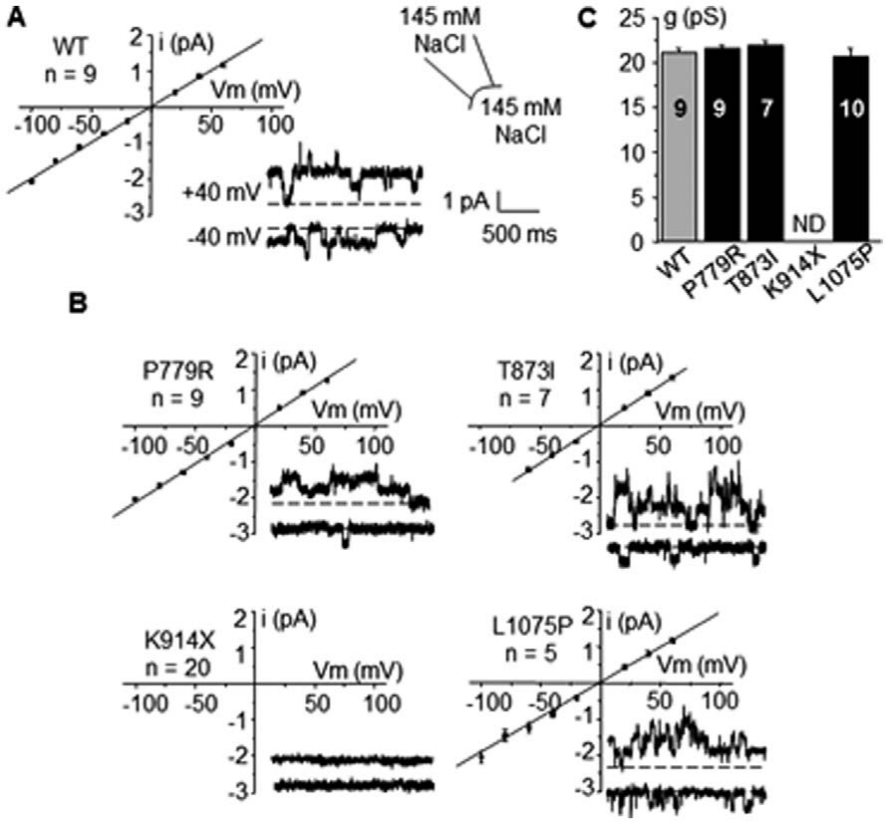
Single channel currents. Inside-out single channel currents for WT and mutants TRPM4. Representative recordings at Vm = +40 and −40 mV and mean current/voltage relationship of WT (**A**) and mutants (**B**). No significant currents were detected for K914X mutant. (**C**): Single channel conductance g of WT and mutants. Mean values for 5 to 9 experiments. ND: not determined.

In HEK-293 cells stably expressing wild type TRPM4, a classical TRPM4 single current was detected in the inside-out configuration (figure 4 A) with a linear current-voltage relationship, providing a single channel conductance of 21.1±0.6 pS (n = 9) in accordance with previous reports [18], [20]. Inside-out patches from p.Lys914X mutant transfected cells did not exhibit any detectable current (n = 20) (figure 4 B). Thus, this mutant was not further investigated. All other mutants provided detectable currents with a current-voltage relationship satisfactorily fitted to a linear regression (figure 4 B) providing single channel conductances g similar to WT (figure 4 C).

On single channel traces provided for WT and each mutant in figure 4, channel activity was higher at Vm = +40 mV than at −40 mV, indicating a channel sensitivity to voltage, a TRPM4 fingerprint.

#### Anion to cation permeability ratio

**Figure 5:**
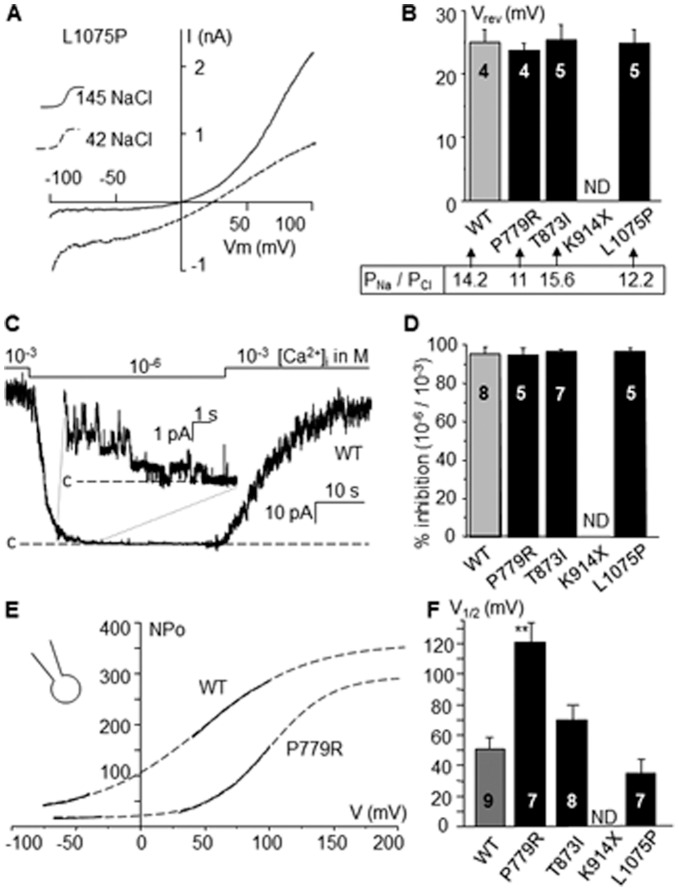
Na^+^/Cl^−^ permeability ratio and channels regulation. (**A**): P_Na_/P_Cl_ permeability ratio was estimated by changing the 145 mM NaCl solution to a 42 mM NaCl solution to measure the shift of the current-voltage relationship. Voltage ramp protocol from Vm = −100 to +100 mV was applied as showed for L1075P. (**B**): Reversal potential (V_rev_) was estimated for WT and mutants as showed in A. P_Na_/P_Cl_ was calculated according to the GHK equation. Similar results were obtained for mutants and WT. (**C**): Effect of [Ca^2+^]_i_ on unitary channel activity was evaluated at Vm = +40 mV by reducing [Ca^2+^]_i_ from 10^−3^ M to 10**^−^** ^6 ^M. A representative trace is provided for WT. Magnification allows observing single-channel currents. Label “c” indicates the current level corresponding to the closed state of all channels. (**D**): Mean % of inhibition of channel activity with [Ca^2+^]_i_  = 10**^−^** ^6 ^M compare to 10**^−^** ^3^ M for WT and mutants. No significant differences were detected. (**E**): Channel sensitivity to voltage was evaluated in the whole-cell configuration by estimating NPo in function of voltage for each mutant during ramp protocols (see proceedings description in the text) as showed for WT and P779R. (**F**): Mean voltage for half maximal activity (V_1/2_) estimated from traces as showed in E. P779R exhibited a significant increase in V_1/2_. ** = significantly different from WT (p<0.01). Numbers in bars = number of experiments.

The anion to cation permeability ratio was investigated in inside-out patches. Reducing internal NaCl concentration from 145 mM to 42 mM shifted the reversal potential (V_rev_). A representative recording is provided for p.Leu1075Pro (figure 5 A). The reversal potential of 25±2 mV (n = 4) for WT with 42 mM NaCl, corresponds to a permeability ratio P_Na_/P_Cl_ of 14.2 according to the Goldman Hodgkin Katz (GHK) equation. All mutants exhibited similar shifts in V_rev_ indicating no variation in their permeability ratio (figure 5 B).

#### Sensitivity to calcium

Reducing internal calcium concentration from 10**^−^** ^3^ to 10**^−^** ^6^ M suppressed 95.5±3.5% of WT TRPM4 activity (figure 5 C). A similar rapid and reversible decrease was observed for all mutants, (figure 5 D), indicating that mutants conserve the TRPM4 sensitivity to calcium.

#### Sensitivity to voltage

The normalized open probability (NP_o_) for each mutant was estimated during ramp protocols, considering that the single channel conductance is linear in all cases (see figure 4). NP_o_ was estimated in function of voltage by transforming the whole-cell current-voltage relationship (I/V) to an NP_o_/V curve using the relation NP_o_ = I/gV (figure 5 E). The curve was then fitted to a Boltzman equation and voltage for half maximal activation (V_1/2_) was determined (figure 5 F). V_1/2_ was significantly increased for p.Pro779Arg compared to WT, while other mutants did not exhibited significant changes.

#### Activation time

Activation time of the current was determined using a pulse protocol from V_m_  = 0 to +80 mV (figure S1 A). No significant differences were seen between mutants and WT (figure S1 B).

#### Channel detection in the inside-out configuration

Channel expression was evaluated in the inside-out configuration by estimating the maximal number of channels opened at V_m_ = +40 mV with 10**^−^** ^3^ M [Ca^2+^]_i_. As shown in figure S2, it was observed a significant decrease in the number of active channels for p.Pro779Arg compared to WT and as mentioned before, no active channels were detected for p.Lys914X. Other mutants did not shown significant variation with WT.

#### Total and cell surface expression of TRPM4 mutant channels

**Figure 6:**
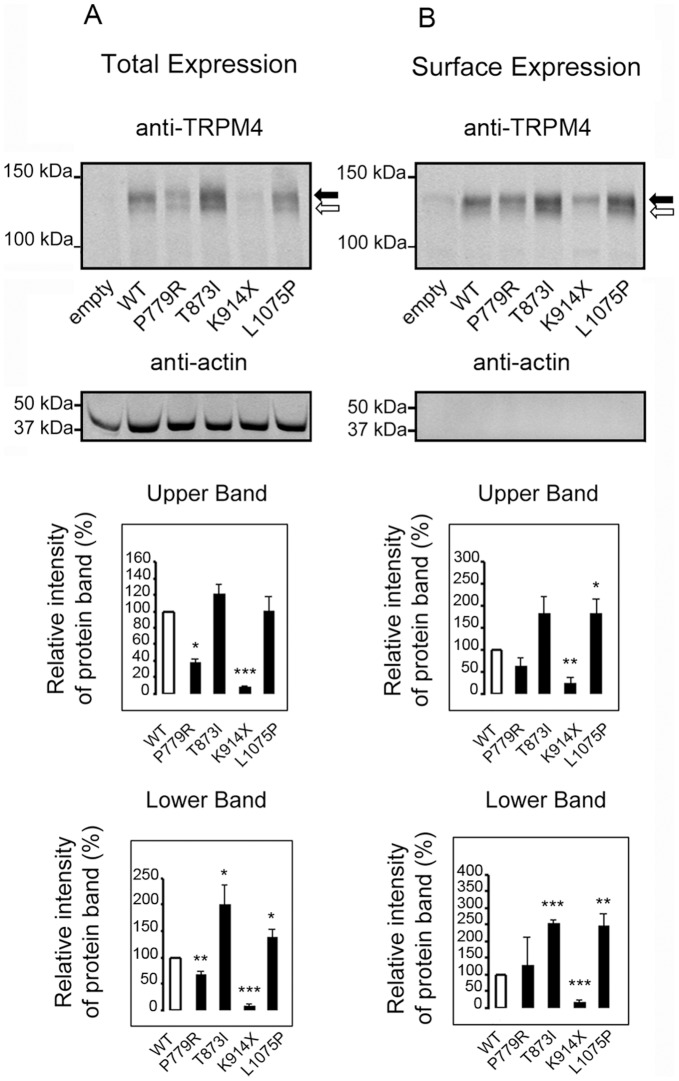
Expression of TRPM4 channel in whole cell extracts and plasma membrane fraction. Several TRPM4 mutants show an alteration of protein expression at a total level as well as at the cell surface. (**A**) Whole cell lysates from HEK-293 cells transfected with TRPM4 constructs used as input, representing the total expression of TRPM4 protein. Quantification of the double bands, presumably fully and core glycosylated forms of TRPM4, black and white arrrows, respectively, is shown on the bottom panels. (**B**) The biotinylated fractions from the same transfection represent the amount of TRPM4 expressed at the cell surface. Quantification of the double bands is shown on the bottom panels. n = 3. *p<0.05, **p<0.01, ***p<0.001. Error bar: standard error of the mean.

To test whether the BrS mutations altered the cellular and cell surface expression level of TRPM4 channels, WT and TRPM4 mutants were transiently transfected in HEK-293 cells. Forty-eight hours post-transfection, the expression of TRPM4 channels at the total protein level and at the cell surface was assessed by quantitative Western blots (figure 6 and figure S3). Under our migration conditions, two distinct bands representing fully and core glycosylated forms of TRPM4 were observed. All mutants showed a significantly altered expression of TRPM4. The p.Pro779Arg and p.Lys914X mutants showed a decreased of total expression whereas p.Lys914X was comparable to background level. With an anti-HA antibody, a shorter band was visible in the total and the surface expression demonstrating that p.Lys914X results in the production of a truncated protein (figure S4). By contrast, the p.Thr873Ile mutant showed a significant increase in the core glycosylated form (lower band) of total and surface expressions whereas the p.Leu1075Pro had a significant surface expression increase.

## Discussion

Here, we present a series of genetic variants found in a large cohort of spontaneous or drug-challenged type 1 BrS patients. Among the 14 *TRPM4* variants, 3 were considered as polymorphisms. By contrast, the 11 remaining variants are presumably pathogenic mutations. Among these 11 mutations, 5 are totally absent from the very large control cohorts (more than 6 500 individuals), whereas 4 others have a statistically higher prevalence in the BrS cohort than in the control cohorts. As an autosomal condition with incomplete penetrance, it is not surprising that genetic variants resulting in BrS or predisposing to BrS might be found in large control cohorts. It is important to note that the size of the BrS and control cohorts allow us to use statistical tests to assess a difference of variant prevalence between both groups.

The variants p.A432T and p.G844D were previously found in families with autosomal dominant cardiac conduction blocks [16]. None of the mutation carriers in the conduction block families had (even retrospectively) an ECG suggestive of a BrS [16]. Interestingly, in this series of 20 BrS cases with TRPM4 mutations or predisposing factors, 18 patients had a conduction block. The 2 patients with no widening of QRS had a rSr’ complex. This observation suggests that TRPM4 mutation screening should be considered in BrS when a widening of QRS or a rSr’ complex is observed, a condition which not rare in BrS cases. Similar to the families with cardiac conduction blocks, no cases of left bundle branch block were observed.

The genetics of BrS is complex with incomplete penetrance and phenocopies [7]. In addition, the causality link of the *SCN5A* variants has been debated [7].It is possible that some BrS cases of the present cohort carry mutations in one or several of the 10 other BrS susceptibility genes. The prevalence of mutations in these 10 genes is low compared to the prevalence of *SCN5A* mutation which accounts for a maximum of 30% [5]. The sum of the prevalences of all the so far published gene mutations does exceed 50% which suggests that about half of BrS cases have mutation in up to now undiscovered genes. In conclusion, a *TRPM4* mutation was found in 9 of 331 BrS patient (2.7%), and a mutation or a predisposing factor was found in 11 patients of the same cohort (3.3%). This suggests that *TRPM4* accounts for a small percentage of BrS, and may thus explain that no mutations were found in a much smaller series [17].

Among the 4 mutants that were further studied, the most remarkable mutant is p.Lys914X since, as expected, it did not produce any current. On Western blots, this mutant gave bands of the same intensity as mock transfected HEK 293 cells consistent with endogenous expression of TRPM4 in this cell line [20] (Amarouch et al., in preparation). Nevertheless, using an anti-HA antibody (the HA tag is located at the N terminus of transfected TRPM4), we could detect a clear band corresponding to the truncated protein in whole cell extract and in the plasma membrane. Since the nonsense mutation is at the end of the fourth trans-membrane domain, this protein lacks the 2 last trans-membrane domains and the extra-cellular segment that forms the pore region. Hence, it not surprising that no current could be recorded. It should be stressed though that in our experimental setting with stable transformed cell lines, we could not evaluate the consequence of the combined expression of wild type and mutant TRPM4 channels. Therefore, we cannot be certain that the K914X variant is a dominant variant.

A decrease in current density was observed for the p.Pro779Arg mutant. Pro779 is located in the second trans-membrane domain and changes a hydrophobic residue to a non-hydrophobic residue. The decrease in current density is probably due to a combination of the decrease in channel expression evidenced by electrophysiological and biochemical assays, and by a modification of channel voltage sensitivity leading to decreased current at physiological voltages.

The variants p.Thr873Ile and p.Leu1075Pro showed no alteration of whole-cell current, single channel properties, and TRPM4 channel regulation. Western blots showed an increased surface expression although the full glycosylated expression of p.Thr873Ile did not reach a statistical threshold.

The mechanisms linking TRPM4 functional alterations and ECG perturbations observed in BrS remain to be clarified. The main perturbation characteristic of the pathology is the ST-segment elevation observed in ECGs. Two models have been proposed to account for the ST segment elevation in BrS: the repolarizing disorder and the depolarizing disorder hypothesis [21].

The repolarizing model is mainly based on the transmural voltage gradient caused by heterogeneity in action potential (AP) plateau among cells spanning the ventricular wall. Change in AP dome depends on modifications of currents activated during the early repolarization and plateau phases of the AP, mainly I_to_, I_Na_ and I_Ca_. TRPM4 may participate in AP shape by promoting the plateau. Due to its non-selective cationic selectivity, TRPM4 activation drives the membrane potential to 0 mV by conducting an outward repolarizing K^+^ current at positive voltages, but an inward depolarizing Na^+^ current at negative voltages. Because TRPM4 is activated by internal Ca^2+^, it is more likely to activate during the plateau phase when internal Ca^2+^ increased and thus counteracts repolarizing K^+^ currents. Thereby, modifications of TRPM4 expression by mutations would change AP dome. This might explain the effect of mutants leading to increased expression but not reduced expression since TRPM4 is only weakly expressed in normal mammalian ventricle [15], [16], [22].

On the other hand, the depolarizing model depends more on conduction delay in the right ventricular outflow tract (RVOT) than differences in AP shape. RVOT perturbations are presented as a substrate site for ventricular tachyarrhythmias [23], [24]. While TRPM4 is poorly expressed in mammalian ventricle, it is more expressed in nodal tissue [15], [16], [25]. Interestingly, the embryologic origin of RVOT is similar to those of atrioventricular regions [21] but different from those of ventricles. According to this, TRPM4 might be abundantly expressed in RVOT. It can be speculated that in analogy to the phenomenon of supernormal excitability and conduction [26], both a gain-of-function and loss-of-function of TRPM4 channels may lead to conduction slowing by reducing the availability of Nav1.5 sodium channels. A gain-of-function may depolarize the resting membrane potential and thus inactivate sodium channels, while a loss-of-function could lead to a hyperpolarization of the membrane potential, and so reduce cellular excitability and conduction. These putative mechanisms of action may be the basis of the observed phenotypic overlap found in patients with *SCN5A* loss-of-function variants and *TRPM4* variants.

In addition to direct effects of *TRPM4* mutations cardiac excitability, one has to consider that these mutants may also have complex effects leading to BrS related to the fact that TRPM4 is expressed in a variety of tissues [27]. Mutations may influence neuro-hormonal regulation or cardiac development [28].

Altogether, this study suggests a role of TRPM4 in BrS accounting for 2.7 to 6% of cases. In contrast to the first 4 *TRPM4* mutations reported in patients with conduction blocks [15], [16], the electrophysiological consequences of the mutations resulting in BrS is more diverse at least 2 mutations resulting in decreased current density, 2 mutations with no electrophysiological anomalies in this experimental setting, and 2 previously reported mutations with increased current. The complexity of the induced disturbances in channel electrophysiology and trafficking is increased by the genetic heterogeneity of BrS. In particular, further studies are warranted to improve our understanding of the interaction between channels that are permeable to sodium and potassium.

## Supporting Information

Figure S1 **Activation time.** Activation time of the current was determined in the whole-cell configuration using a pulse protocol from Vm  = 0 to +80 mV. Currents were fitted to a double exponential to estimate time for half activation. A: Current trace for WT under a pulse protocol as showed under the trace. B: Mean time for half activation for WT and mutants. No significant differences were seen between mutants and WT.
(TIF)

Figure S2 **Number of channels per patch detected in inside-out configuration.** Mean number of TRPM4 channels detected in each inside-out patch at Vm = +40 mV (pipette and bath: 145 mM NaCl, 10^−3^ M Ca^2+^). No detectable current was observed for K914X. Number of experiments on top of bars.
(TIF)

Figure S3 **Original western blot pictures that were including more mutants than presented in** **figure 6** **.** Panels are from top to bottom: total expression and anti-TRPM4 antibody, total expression and anti-actin antibody; surface expression and anti-TRPM4 antibody; surface expression and anti-actin antibody. Lanes are from left-hand side to right-hand side: empty plasmid, size marker, wild type TRPM4, and the following TRPM4 mutants: L138P, R164W, A432T, G737R, P779R, G844D, T873I, K914X, L941M, L1075P and E7K.
(TIF)

Figure S4 **Original pictures of western blots showing total and surface expression revealed with an anti-HA antibody.** These are the original Western blot pictures that included the same lanes as in figure S3. The method used is slightly different than in western blot of figure 6 and S3 in particular an anti-HA antibody was used instead of an anti-TRPM4 antibody. Note that a shorter band is clearly visible on the L914X mutant line suggesting a truncated TRPM4 mutant present in the total expression but also in the surface expression.
(TIF)
