# Supplementary figures and images for: Molecular Genetics and Functional Anomalies in a Series of 248 Brugada Cases with 11 Mutations in the TRPM4 Channel
Source: PLoS One. 2013 Jan 30;8(1):e54131. doi: 10.1371/journal.pone.0054131 (PMC3559649; doi:10.1371/journal.pone.0054131)

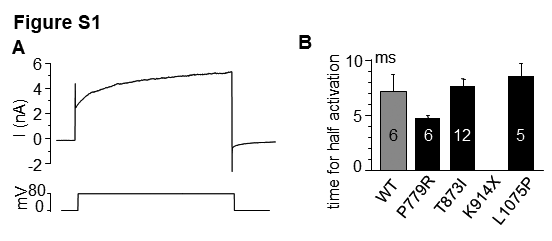

Supplement: Figure S1 — Activation time. Activation time of the current was determined in the whole-cell configuration using a pulse protocol from Vm = 0 to +80 mV. Currents were fitted to a double exponential to estimate time for half activation. A: Current trace for WT under a pulse protocol as showed under the trace. B: Mean time for half activation for WT and mutants. No significant differences were seen between mutants and WT. (TIF) [file pone.0054131.s001.tif]

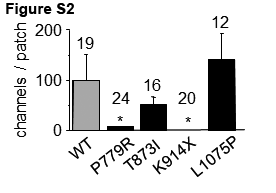

Supplement: Figure S2 — Number of channels per patch detected in inside-out configuration. Mean number of TRPM4 channels detected in each inside-out patch at Vm = +40 mV (pipette and bath: 145 mM NaCl, 10−3 M Ca2+). No detectable current was observed for K914X. Number of experiments on top of bars. (TIF) [file pone.0054131.s002.tif]

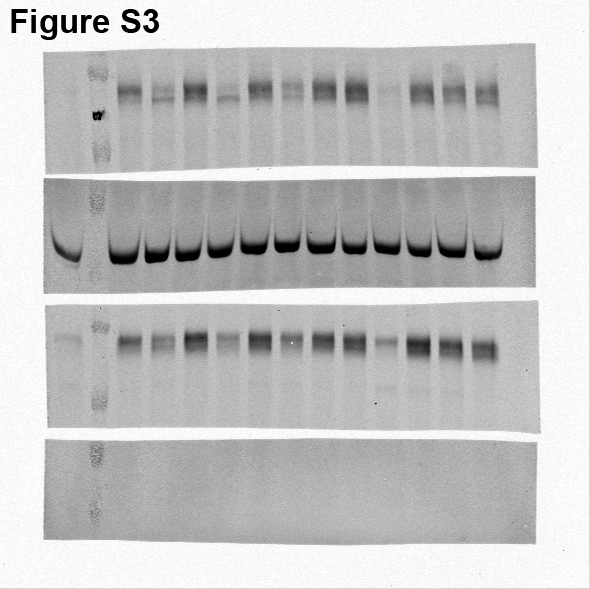

Supplement: Figure S3 — Original western blot pictures that were including more mutants than presented in figure 6 . Panels are from top to bottom: total expression and anti-TRPM4 antibody, total expression and anti-actin antibody; surface expression and anti-TRPM4 antibody; surface expression and anti-actin antibody. Lanes are from left-hand side to right-hand side: empty plasmid, size marker, wild type TRPM4, and the following TRPM4 mutants: L138P, R164W, A432T, G737R, P779R, G844D, T873I, K914X, L941M, L1075P and E7K. (TIF) [file pone.0054131.s003.tif]

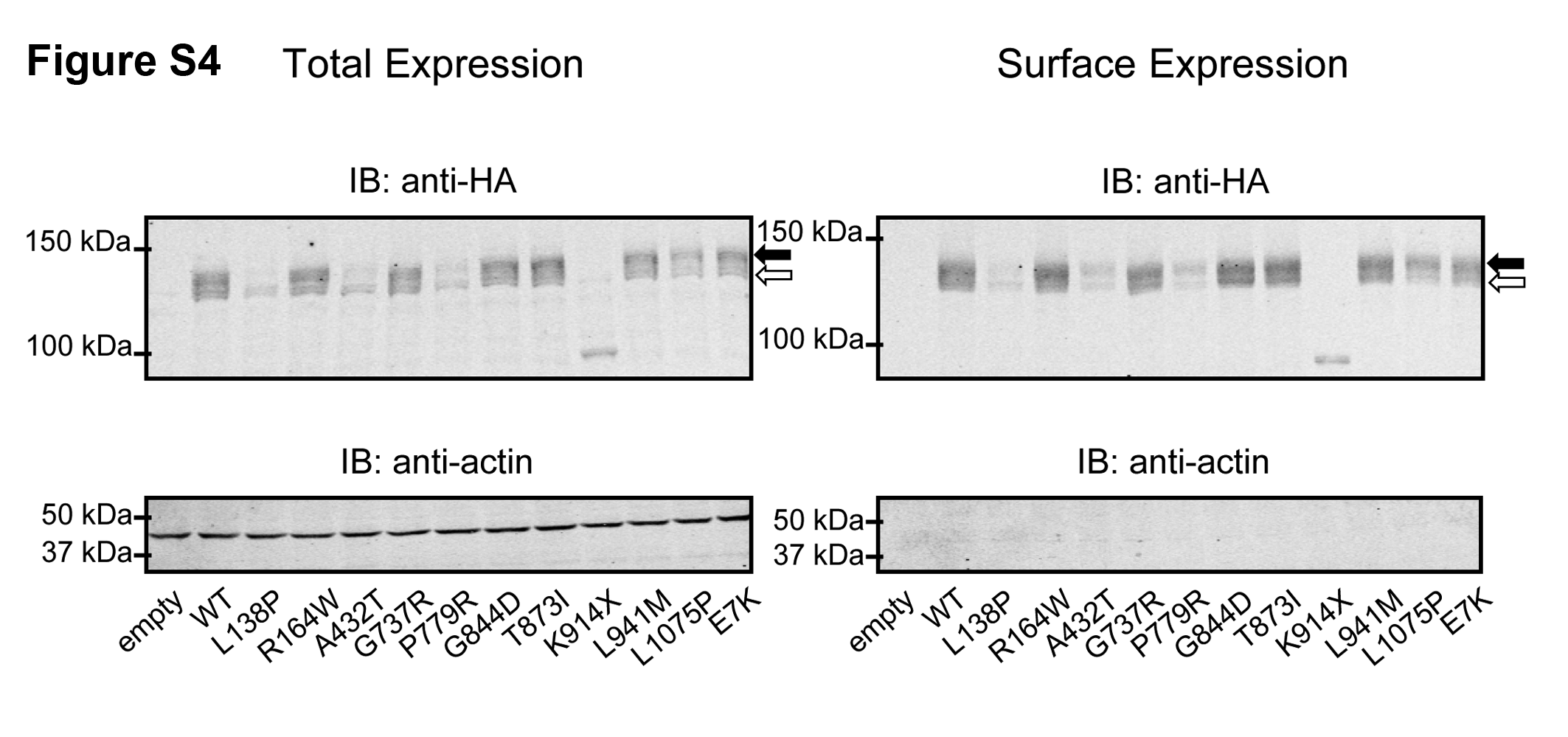

Supplement: Figure S4 — Original pictures of western blots showing total and surface expression revealed with an anti-HA antibody. These are the original Western blot pictures that included the same lanes as in figure S3. The method used is slightly different than in western blot of figure 6 and S3 in particular an anti-HA antibody was used instead of an anti-TRPM4 antibody. Note that a shorter band is clearly visible on the L914X mutant line suggesting a truncated TRPM4 mutant present in the total expression but also in the surface expression. (TIF) [file pone.0054131.s004.tif]
